# Assessing the lack of diversity in genetics research across neurodegenerative diseases: A systematic review of the GWAS Catalog and literature

**DOI:** 10.1002/alz.13873

**Published:** 2024-06-21

**Authors:** Caroline Jonson, Kristin S. Levine, Julie Lake, Linnea Hertslet, Lietsel Jones, Dhairya Patel, Jeff Kim, Sara Bandres‐Ciga, Nancy Terry, Ignacio F. Mata, Cornelis Blauwendraat, Andrew B. Singleton, Mike A. Nalls, Jennifer S. Yokoyama, Hampton L. Leonard

**Affiliations:** ^1^ Center for Alzheimer's and Related Dementias National Institutes of Health Bethesda Maryland USA; ^2^ DataTecnica LLC Washington District of Columbia USA; ^3^ Pharmaceutical Sciences and Pharmacogenomics Graduate Program University of California San Francisco California USA; ^4^ Memory and Aging Center Department of Neurology Weill Institute for Neurosciences University of California San Francisco California USA; ^5^ Laboratory of Neurogenetics National Institutes on Aging National Institutes of Health Bethesda Maryland USA; ^6^ Integrative Neurogenomics Unit Laboratory of Neurogenetics National Institute on Aging National Institutes of Health Bethesda Maryland USA; ^7^ Division of Library Services Office of Research Services National Institutes of Health Bethesda Maryland USA; ^8^ Genomic Medicine Institute, Lerner Research Institute, Genomic Medicine Cleveland Clinic Foundation Cleveland Ohio USA; ^9^ Department of Radiology and Biomedical Imaging University of California, San Francisco San Francisco California USA; ^10^ German Center for Neurodegenerative Diseases (DZNE) Tübingen Germany

**Keywords:** Alzheimer's disease, amyotrophic lateral sclerosis, ancestral diversity, genetic research disparities, genome‐wide association study, multi‐ancestry cohorts, neurodegenerative diseases, Parkinson's disease, population genetics, precision medicine

## Abstract

The under‐representation of non‐European cohorts in neurodegenerative disease genome‐wide association studies (GWAS) hampers precision medicine efforts. Despite the inherent genetic and phenotypic diversity in these diseases, GWAS research consistently exhibits a disproportionate emphasis on participants of European ancestry. This study reviews GWAS up to 2022, focusing on non‐European or multi‐ancestry neurodegeneration studies. We conducted a systematic review of GWAS results and publications up to 2022, focusing on non‐European or multi‐ancestry neurodegeneration studies. Rigorous article inclusion and quality assessment methods were employed. Of 123 neurodegenerative disease (NDD) GWAS reviewed, 82% predominantly featured European ancestry participants. A single European study identified over 90 risk loci, compared to a total of 50 novel loci in identified in all non‐European or multi‐ancestry studies. Notably, only six of the loci have been replicated. The significant under‐representation of non‐European ancestries in NDD GWAS hinders comprehensive genetic understanding. Prioritizing genomic diversity in future research is crucial for advancing NDD therapies and understanding.

**Highlights:**

- Eighty‐two percent of neurodegenerative genome‐wide association studies (GWAS) focus on Europeans.
- Only 6 of 50 novel neurodegenerative disease (NDD) genetic loci have been replicated.
- Lack of diversity significantly hampers understanding of NDDs.
- Increasing diversity in NDD genetic research is urgently required.
- New initiatives are aiming to enhance diversity in NDD research.

## 1 INTRODUCTION

Genome‐wide association studies (GWAS) have shown a significant bias toward individuals of European ancestry, despite comprising only 16% of the global population.^1^ This under‐representation issue is particularly salient in the realm of neurodegenerative disease (NDD) studies. For instance, although a recent Alzheimer's disease (AD) GWAS including ≈800,000 individuals of European descent identified 75 disease‐associated loci,^2^ no GWAS studies on AD currently exist for Admixed American or Native American populations. Similarly, Parkinson's disease (PD) research exhibits a glaring imbalance, with Black individuals included in just ≈4% of published PD studies.^3^

The prevalence of NDDs varies significantly among global populations and racial/ethnic groups. This warrants a critical examination of the disparity in genetic research efforts over time. In this article, we present a systematic review spanning 2012 through 2022, focusing on NDD GWAS research.

Our analysis encompasses common NDDs such as AD, PD, and amyotrophic lateral sclerosis (ALS), as well as less common atypical dementias. Our objective is to quantify the disparity in participant recruitment for genetic studies, shed light on genetic findings in under‐represented populations, and discuss ongoing initiatives aimed at addressing this pervasive issue.

## 2 METHODS

### 2.1 Search strategy

The systematic review was conducted in two phases on seven of the most common NDDs: Alzheimer's disease (or AD), Parkinson's disease (or PD), amyotrophic lateral sclerosis (or ALS), multiple sclerosis (MS), frontotemporal dementia (FTD), myasthenia gravis (MG), Lewy body dementia (LBD), and vascular dementia (VaD). First, we reviewed the GWAS Catalog; then, because the GWAS Catalog does not include all GWAS studies, we performed a formal literature review in collaboration with the National Library of Medicine (NLM). The keywords used in both searches are included in Table S1. The GWAS Catalog uses precise indexing terms, and our targeted keyword strategy was intended to maximize the retrieval of studies, thereby ensuring a comprehensive review. By including specific onset‐related terms, we aimed to cover all types of NDD GWAS research relevant to our review, acknowledging that the correct keywords can significantly enhance study inclusion.

Results from both the GWAS Catalog and the NLM search were uploaded to Covidence,^4^ a web‐based software platform, for further review. We removed duplicate studies and any studies published before 2012 or after 2022.

All studies were filtered to include only genome‐wide associations examining neurological disease risk factors, family history of disease, disease progression, age at onset, or survival genome‐wide, excluding exome‐wide studies and those that focused on a targeted set of single nucleotide polymorphisms (SNPs) or genetic loci. Studies investigating disease subtypes, biomarkers, or non–English‐language studies, and those investigating only rare or structural variation were also excluded.

Studies were assessed for eligibility by two independent reviewers and all conflicts were resolved by a third independent reviewer. Publication date, phenotype, and cohort information were extracted from each publication. If multiple phenotypes of interest were analyzed in the same study, information was included in both phenotype categories. The number of samples per ancestry was extracted manually from each study. We looked at seven ancestry groupings: European (EUR), East Asian (EAS), Middle‐Eastern (MDE), African (AFR), African American and Caribbean (AAC), Latino and Indigenous Americas populations (AMR), and South Asian (SAS). If more than one ancestry group was present, the study was labeled multi‐ancestry (MULTI). In this article, multi‐ancestry refers to a combination of ancestral groups rather than individuals who are mixtures of ancestral backgrounds. A Preferred Reporting Items for Systematic Reviews and Meta‐Analyses (PRISMA) diagram of our filtering process can be found in Figure S1. All 123 studies passing our filters can be found in Table S2.

Finally, results were examined manually for all studies passing implemented filtering methods, flagging novel loci discovered in non‐European or multi‐ancestry populations with a *p*‐value below 5E‐8. We checked for replication of novel loci using the Open Targets platform.

## 3 RESULTS

### 3.1 Search results

**FIGURE 1 alz13873-fig-0001:**
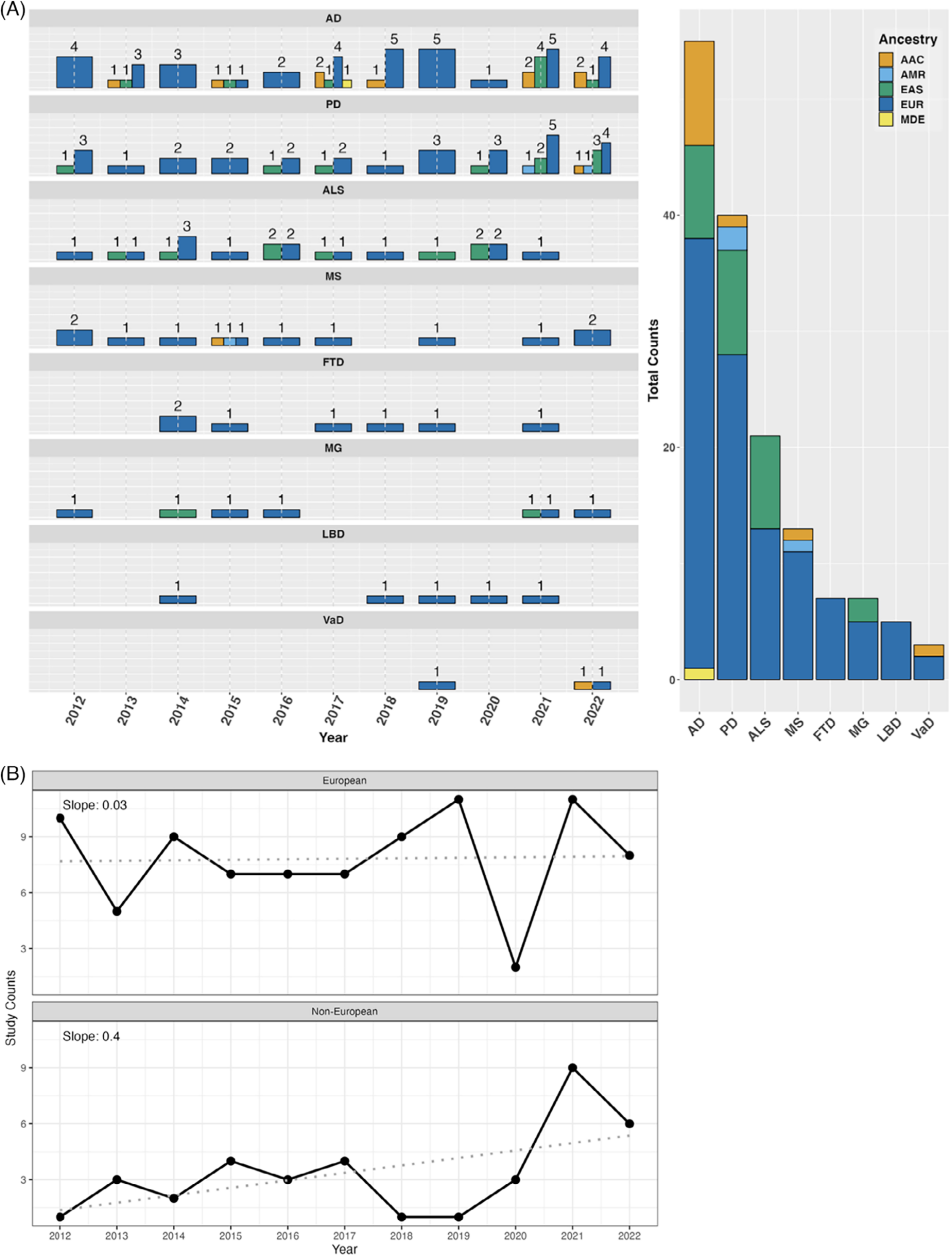
Number of studies over time from 2012 to 2022. (A) Bar plot of study counts by NDD (left) with cumulative counts for each ancestry (right). Data in this figure include both single and multi‐ancestry studies. (B) Time series of the annual study counts in European and non‐European populations from 2012 to 2022. The slope from a linear regression is also displayed to highlight the rate of change in the number of study counts over time. AAC, African American and Caribbean; AD, Alzheimer's disease; AFR, African; ALS, amyotrophic lateral sclerosis; AMR, Latino and Indigenous Americas populations; EAS, East Asian; EUR, European; FTD, frontotemporal dementia; LBD, Lewy body dementia; MDE, Middle‐Eastern; MG, myasthenia gravis; MS, multiple sclerosis; NDD, neurodegenerative disease; PD, Parkinson's disease; SAS, South Asian; VaD, vascular dementia.

We identified **123** eligible GWAS studies. Unsurprisingly, we found that European populations were over‐represented in GWAS pertaining to NDDs. When non‐European populations were included, the sample sizes were on average 15× smaller than the European ancestry samples included in the same disease category. The under‐representation of non‐European populations was particularly evident among the less common NDDs, including LBD, FTD, and VaD, where we did not identify any non‐European or multi‐ancestry GWAS studies using the outlined search methods. We have summarized the lack of diversity in genetic studies of NDDs in Table 1 and Figure 1.

**TABLE 1:** Largest GWAS sample size by NDD and ancestry for single and multi‐ancestry studies.

NDD	Ancestry	Author	Year	Total samples (% non‐European*)	*N*_proxycases_ *N*_cases_ *N*_controls_	Minor Allele Frequency threshold (MAF)	Study (DOI)
AD	AAC	Sherva	2022	75,058	6641 4012 64,405	1%	African ancestry GWAS of dementia in a large military cohort identifies significant risk loci (10.1038/s41380‐022‐01890‐3)
	EAS	Hirano	2015	17,031	0 1827 15,204	1%	A genome‐wide association study of late‐onset Alzheimer's disease in a Japanese population (10.1097/YPG.0000000000000090)
	EUR	Wightman	2021	1,126,563	46,613 43,725 1,036,225	∼0.06%	A genome‐wide association study with 1,126,563 individuals identifies new risk loci for Alzheimer's disease (10.1038/s41588‐021‐00921‐z)
	MULTI	Lake	2022	644,188 (2.83%)	46,828 54,233 543,127	1%	Multi‐ancestry meta‐analysis and fine‐mapping in Alzheimer's disease (10.1038/s41380‐023‐02089‐w)
	AMR, MDE			NO STUDIES			
PD	AMR	Loesch	2021	1497	0 807 690	1%	Characterizing the genetic architecture of Parkinson's disease in Latinos (10.1002/ana.26153)
	EAS	Foo	2017	14,006	0 779 13,227	1%	Genome‐wide association study of Parkinson's disease in East Asians (10.1093/hmg/ddw379)
	EUR	Nalls	2019	1,474,097	18,618 37,688 1,417,791	1% & 5%	Identification of novel risk loci, causal insights, and heritable risk for Parkinson's disease: a meta‐analysis of genome‐wide association studies (10.1016/S1474‐4422(19)30320‐5)
	MULTI	Kim	2022	2,525,730 (38.12%)	18,618 49,049 2,458,063	0.1%	Multi‐ancestry genome‐wide meta‐analysis in Parkinson's disease (10.1038/s41588‐023‐01584‐8)
	AAC, MDE	NO STUDIES					
ALS	EAS	Wei	2019	4727	0 700 4027	5%	Identification of TYW3/CRYZ and FGD4 as susceptibility genes for amyotrophic lateral sclerosis (10.1212/NXG.0000000000000375)
	EUR	van Rheenen	2016	41,398	0 15,156 26,242	1%	Genome‐wide association analyses identify new risk variants and the genetic architecture of amyotrophic lateral sclerosis (10.1038/ng.3622)
	MULTI	van Rheenen	2021	152,268 (12.00%)	0 29,612 122,656	0.1%	Common and rare variant association analyses in amyotrophic lateral sclerosis identify 15 risk loci with distinct genetic architectures and neuron‐specific biology (10.1038/s41588‐021‐00973‐1)
	AAC, AMR, MDE	NO STUDIES					
MS	AAC	Isobe	2015	2319	0 803 1516	1%	An ImmunoChip study of multiple sclerosis risk in African Americans (10.1093/brain/awv078)
	AMR	Ordoñez	2015	161	0 29 132	1%	Genome wide admixture study in Mexican Mestizos with multiple sclerosis (10.1016/j.clineuro.2014.11.026)
	EUR	Patsopoulos	2019	115,803	0 47,429 68,374	1%	Multiple sclerosis genomic map implicates peripheral immune cells and microglia in susceptibility (10.1126/science.aav7188)
	EAS, MDE, MULTI	NO STUDIES					
FTD	EUR	Ferrari	2014	12,928	0 3526 9402		Frontotemporal dementia and its subtypes: a genome‐wide association study (10.1016/S1474‐4422(14)70065‐1)
	AAC, AMR, EAS, MDE, MULTI	NO STUDIES					
MG	EAS	Na	2014	259	0 109 150	1%	Whole‐genome analysis in Korean patients with autoimmune myasthenia gravis (10.3349/ymj.2014.55.3.660)
	EUR	Chia	2022	45,675	0 2227 43,448	0.1%	Identification of genetic risk loci and prioritization of genes and pathways for myasthenia gravis: a genome‐wide association study (10.1073/pnas.2108672119)
	MULTI	Sakaue	2021	533,853 (33.48 %)	0 278 533,575	0.01%	A cross‐population atlas of genetic associations for 220 human phenotypes (10.1038/s41588‐021‐00931‐x)
	AAC, AMR, MDE	NO STUDIES					
LBD	EUR	Chia	2021	7372	0 2981 4391	1%	Genome sequencing analysis identifies new loci associated with Lewy body dementia and provides insights into its genetic architecture (10.1038/s41588‐021‐00785‐3)
	AAC, AMR, EAS, MDE, MULTI	NO STUDIES					
VaD	EUR	Moreno‐Grau	2019	4830	0 1541 3289	1%	Genome‐wide association analysis of dementia and its clinical endophenotypes reveal novel loci associated with Alzheimer's disease and three causality networks: The GR@ACE project (10.1016/j.jalz.2019.06.4950)
	MULTI	Fongang	2022	482,088 (2.40%)	0 4138 477,950	1%	A meta‐analysis of genome‐wide association studies identifies new genetic loci associated with all‐cause and vascular dementia (10.1002/alz.056081)
	AAC, AMR,EAS, MDE	NO STUDIES					

*Note*: Minor allele frequency (MAF) thresholds are included as inappropriate selection of MAF thresholds can lead to spurious findings and misleading reports of heterogeneity.

Abbreviations: AAC, African American and Caribbean; AD, Alzheimer's disease; AFR, African; ALS, amyotrophic lateral sclerosis; AMR, Latino and Indigenous Americas populations; EAS, East Asian; EUR, European; FTD, frontotemporal dementia; GWAS, genome‐wide association studies; LBD, Lewy body dementia; MDE, Middle Eastern; MG, myasthenia gravis; MS, multiple sclerosis; NDD, neurodegenerative disease; PD, Parkinson's disease; SAS, South Asian; VaD, vascular dementia.

*If applicable.

We found 50 novel NDD loci that were identified in non‐European or multi‐ancestry populations (Table 2). Of these **50** loci, **24** were found in multi‐ancestry studies, **23** were found in East Asian studies, and only **3** were found in other populations (AAC and AMR). No loci were discovered in AFR, MDE, or SAS ancestries. Recent studies that combined individuals of multiple ancestries by using standard random‐effects and some custom meta‐analytic techniques^5^ have succeeded in identifying novel disease loci that reach genome‐wide significance, including two novel AD loci^6^ and 12 novel PD loci.^7^ However, these studies leverage existing European sample sizes as a backbone for much of the statistical power needed for discovery.

**TABLE 2 alz13873-tbl-0002:** Genome‐wide significant (*p*‐value < 5E‐8) novel loci nominated in non‐European populations or multi‐ancestry studies.

NDD	Author	Year	Study	Ancestry	Nominated loci	*p*‐value	Replicated (open targets)
AD	Tosto	2015	F‐box/LRR‐repeat protein 7 is genetically associated with Alzheimer's disease (10.1002/acn3.223)	AMR	*FBXL7*	6.19E‐09	no
AD	Mez	2017	Two novel loci, COBL and SLC10A2, for Alzheimer's disease in African Americans (10.1016/j.jalz.2016.09.002)	AAC	*COBL*	3.80E‐08	no
					*SLC10A2*	4.60E‐08	no
AD	Jia	2021	Prediction of Alzheimer's disease using multi‐variants from a Chinese genome‐wide association study (10.1093/brain/awaa364)	EAS	*RHOBTB3/GLRX*	3.07E‐19	no
					*CTC/278L1.1* (rs6859823)	2.49E‐23	no
					*CTD/2506J14.1* (rs234434)	1.35E‐67	no
					*CHODL*	4.81E‐09	no
AD	Kang	2021	Potential Novel Genes for Late‐Onset Alzheimer's Disease in East‐Asian Descent Identified by APOE‐Stratified Genome‐Wide Association Study (10.3233/JAD‐210145)	EAS	*CACNA1A*	2.49E‐08	no
					*LRIG1*	1.51E‐08	no
AD	Shigemizu	2021	Ethnic and trans‐ethnic genome‐wide association studies identify new loci influencing Japanese Alzheimer's disease risk (10.1038/s41398‐021‐01272‐3)	EAS	*FAM47E*	5.34E‐09	no
				MULTI	*OR2B2*	2.14E‐08	no
AD	Miyashita	2013	SORL1 is genetically associated with late‐onset Alzheimer's disease in Japanese, Koreans and Caucasians (10.1371/journal.pone.0058618)	MULTI	*SORL1*	1.04E‐08	Schwartzentruber J (2021), Wightman DP (2021), Marioni RE (2018), Jansen IE (2019), Moreno‐Grau S (2019)
AD	Lake	2022*	Multi‐ancestry meta‐analysis and fine‐mapping in Alzheimer's Disease (10.1038/s41380‐023‐02089‐w)	MULTI	*TRANK1*	3.49E‐08	no
					*VWA5B2*	3.75E‐08	no
ALS	Deng	2013	Genome‐wide association analyses in Han Chinese identify two new susceptibility loci for amyotrophic lateral sclerosis (10.1038/ng.2627)	EAS	*CAMK1G*	2.92E‐08	Wei L (2019)
					*CABIN1/SUSD2*	2.35E‐09	Wei L (2019)
ALS	Xie	2014	Genome‐wide association study combining pathway analysis for typical sporadic amyotrophic lateral sclerosis in Chinese Han populations (10.1016/j.neurobiolaging.2014.01.014)	EAS	*INPP5B*	2.24E‐08	no
					*IQCF5/IQCF1*	2.06E‐09	no
					*ITGA9*	2.55E‐08	no
					*PFKP*	2.46E‐09	no
					*MYO18B*	2.28E‐10	no
					*ALCAM*	4.00E‐08	no
					*OPCML*	8.43E‐09	no
					*GPR133*	8.45E‐10	no
ALS	Wei	2019	Identification of TYW3/CRYZ and FGD4 as susceptibility genes for amyotrophic lateral sclerosis (10.1212/NXG.0000000000000375)	EAS	*TYW3/CRYZ*	2.10E‐14	no
ALS	Benyamin	2017	Cross‐ethnic meta‐analysis identifies association of the GPX3‐TNIP1 locus with amyotrophic lateral sclerosis (10.1038/s41467‐017‐00471‐1)	MULTI	*FGD4*	5.19E‐09	no
ALS	Benyamin	2017	Cross‐ethnic meta‐analysis identifies association of the GPX3‐TNIP1 locus with amyotrophic lateral sclerosis (10.1038/s41467‐017‐00471‐1)	MULTI	*GPX3/TNIP1*	1.30E‐08	van Rheenen W (2021), Nicolas A (2018)
ALS	Nakamura	2020	A multi‐ethnic meta‐analysis identifies novel genes, including ACSL5, associated with amyotrophic lateral sclerosis (10.1038/s42003‐020‐01251‐2)	MULTI	*ACSL5*	2.97E‐08	Iacoangeli (2020)
ALS	van Rheenen	2021	Common and rare variant association analyses in amyotrophic lateral sclerosis identify 15 risk loci with distinct genetic architectures and neuron‐specific biology (10.1038/s41588‐021‐00973‐1)	MULTI	*SLC9A8/SPATA2*	3.20E‐10	no
PD	Li	2022	Genetic Determinants of Survival in Parkinson's Disease in the Asian Population (10.1002/mds.29069)	EAS	*ERGIC1*	5.60E‐09	no
					*NEK1*	6.90E‐09	no
					*COG3*	1.20E‐08	no
PD	Li	2021	Genetic Modifiers of Age at Onset for Parkinson's Disease in Asians: A Genome‐Wide Association Study (10.1002/mds.28621)	EAS	*PTPRN2*	1.80E‐08	no
PD	Foo	2020	Identification of Risk Loci for Parkinson Disease in Asians and Comparison of Risk Between Asians and Europeans: A Genome‐Wide Association Study (10.1001/jamaneurol.2020.0428)	EAS	*SV2C*	3.48E‐08	no
					*WBSCR17*	2.53E‐08	no
PD	Li	2021	Genetic Modifiers of Age at Onset for Parkinson's Disease in Asians: A Genome‐Wide Association Study (10.1002/mds.28621)	EAS	*NDN/PWRN4*	3.14E‐09	no
PD	Li	2022	Genetic Determinants of Survival in Parkinson's Disease in the Asian Population (10.1002/mds.29069)	EAS	*RPL3*	2.72E‐08	no
PD	Lill	2012	Comprehensive research synopsis and systematic meta‐analyses in Parkinson's disease genetics: The PDGene database (10.1371/journal.pgen.1002548)	MULTI	*ITGA8*	1.30E‐08	Nalls MA (2019), Chang D (2017)
PD	Kim	2022*	Multi‐ancestry genome‐wide meta‐analysis in Parkinson's disease (10.1038/s41588‐023‐01584‐8)	MULTI	*MTF2*	1.15E‐10	no
					*PIK3CA*	1.65E‐10	no
					*ADD1*	4.11E‐09	no
					*SYBU*	3.62E‐09	no
					*IRS2*	2.30E‐09	no
					*USP8*	6.45E‐10	no
					*PIGL*	2.93E‐09	no
					*FASN*	2.61E‐09	no
					*MYLK2*	3.86E‐09	no
					*USP25*	1.12E‐09	no
					*EP300*	3.81E‐09	no
					*PPP6R2*	4.09E‐10	no

*Note*: We found no diverse or multi‐ancestry loci for LBD, FTD, MG, or VaD. *p*‐values are given in parentheses. Nominated loci were determined as the nearest gene or genomic context within 1 B of the significant SNP. We looked in Open Targets to determine replicated loci. The * indicates the study was originally available as a preprint.

Abbreviations: AAC, African American and Caribbean; AD, Alzheimer's disease; ALS, amyotrophic lateral sclerosis; AMR, Latino and Indigenous Americas populations; EAS, East Asian; LBD, Lewy body dementia; NDD, neurodegenerative disease; PD, Parkinson's disease.

Of the 50 novel loci, we were could find replication for only 6 (Table 2). Of those six loci, five were found in multi‐ancestry cohorts, which are predominantly powered by European samples. One locus was found in an East Asian population and later replicated in another East Asian population. Genome wide replication is an issue for non‐European studies, because they are often very small and include all samples available. This further highlights the importance of conducting more and larger non‐European studies. As data sets become larger and more inclusive, the genetic architecture of these diseases may grow and change.

In the following sections, we briefly summarize the results of our systematic review in a disease‐specific manner.

**RESEARCH IN CONTEXT**

1. **Systematic review**: We conducted a systematic review utilizing existing genome‐wide association studies (GWAS) results and publications, curated from the GWAS Catalog and with the National Library of Medicine, to assess the inclusion of diverse ancestry groups in neurodegeneration and neurogenetics studies. We employed rigorous methods for the inclusion of identified articles and quality assessment.
2. **Interpretation**: Our findings underscore the pressing need for increased diversity in neurodegenerative research. The significant under‐representation of non‐European ancestry participants in NDD GWAS limits our understanding of the genetic underpinnings of these diseases.
3. **Future directions**: Our work underscores the need for more inclusive research approaches in neurodegenerative diseases that emphasize multi‐ancestry and non‐European populations to advance precision medicine and develop treatments effective for diverse populations. Our review showed that diverse GWAS in non‐European populations have enhanced power, led to the discovery of new loci, and provided a more comprehensive understanding of NDD genetic architecture beyond just European contexts.

**Alzheimer's Disease**

**Largest European GWAS**: Wightman 2021

**Total samples**: 1,126,563

**Largest multi‐ancestry GWAS**: Lake 2022

**Total samples**: 644,188

**Total non‐European samples**: 18,246

**% non‐European**: 2.83%

**Largest non‐European GWAS**: Sherva 2022

**Ancestry**: AAC

**Total samples**: 75,058

Loci discovered in non‐EUR or multi‐ancestry studies: 14

Loci replicated: 1 (*SORL1*)

The largest AD GWAS of European populations included ≈1.1 million individuals and identified a total of 38 associated loci.^8^ Another recent GWAS included ≈800,000 individuals of European ancestry and identified a total of 75 loci.^2^ The discrepancy between identified loci in these studies could be due to many factors, including differences in neuropathological/diagnostic criteria.^9^

A 2013 GWAS conducted in African Americans replicated an association at *ABCA7* previously identified in European populations. That GWAS found that rs115550680, rare in European populations, was associated with an increased risk for AD in African Americans comparable to the highly pathogenic apolipoprotein E (*APOE*) ε4 variant observed in Europeans.^10^ A 2017 GWAS in African Americans identified two novel loci at *COBL* and *SLC10A2*.^11^ The most extensive African American GWAS to date, drawing from a military cohort of ≈22,000 individuals and a proxy GWAS involving ≈50,000 individuals, identified significant associations with established AD risk genes such as *TREM2*, *CD2AP*, and *ABCA7*. Notably, distinct lead variants were observed in these loci compared to those found in European cohorts.^12^

The only study conducted[Fig alz13873-fig-0001] in Caribbean Hispanic individuals was a 2017 study with 2451 cases and 2063 controls. Investigators found a novel and population specific locus near *FBXL7*.^13^ The lead SNP, rs75002042, is much more common in individuals with African ancestry compared to individuals of European ancestry, with minor allele frequencies[Table alz13873-tbl-0002] around 20% and 0.009%, respectively. This study also replicated six loci previously reported in European populations, including *FRMD4A, CELF1, FERMT2, SLC24A4/RIN3, ABCA7*, and *CD33*.^13^

The largest AD study in East Asian populations was conducted in Japanese participants with 1827 cases and 15,204 controls (discovery + replication),^14^ but authors did not nominate any genome‐wide significant loci. More recent but smaller studies have since been conducted, including a 2021 GWAS in a Chinese cohort that reported four novel loci near *RHOBTB3/GLRX, CTC/278L1.1, CTD/2506J14.1*, and *CHODL* ^15^; a study in Japanese participants that nominated a locus in *FAM47E* ^16^; and a study including both Korean and Japanese participants that nominated two novel loci at *CACNA1A* and *LRIG1*.^17^

Multi‐ancestry studies have nominated additional AD loci; however, these studies still rely on Europeans as the majority population. *SORL1* was first identified as a risk locus for AD in a GWAS that included East Asian and European ancestry populations.^18^ Other multi‐ancestry GWAS replicated loci nominated in previous studies as well as identified *OR2B2*,^16^ *TRANK1*, and *VWA5B2* ^19^ as novel loci for AD.

Although the inclusion of diverse populations in genetic research for AD is arguably better than what is seen for some of the atypical dementias, the largest study size for a non‐European population^12^ was still only 7% of the total sample size for the largest European AD GWAS. Only one locus discovered in a non‐European or multi‐ancestry study has been replicated (*SORL1*).

**Parkinson's Disease**

**Largest European GWAS**: Nalls 2019

**Total samples**: 1,456,306

**Largest multi‐ancestry GWAS**: Kim 2022

**Total samples**: 2,525,730

**Total non‐European samples**: 962,735

**% non‐European**: 38.12%

**Largest non‐European GWAS**: Foo 2017

**Ancestry**: EAS

**Total samples**: 14,006

Loci discovered in non‐EUR or multi‐ancestry studies: 17

Loci replicated: 1 (*ITGA8*)

The largest meta‐GWAS of PD risk in individuals of European ancestry found 90 significant risk signals across 78 genomic regions. The 90 nominated risk variants collectively explain roughly 16%–36% of the heritable risk of non‐monogenic or complex PD.^20^

The largest study in East Asian populations (with exception of a study done in Japan before our review period^21^) was conducted with Han Chinese participants, replicating loci identified previously in European populations including *SNCA*, *LRRK2*, and *MCCC1* in their discovery GWAS of 14,006 participants.^22^ More recent studies in Chinese populations have nominated a locus on *NDN/PWRN4* associated with age at onset and a locus on *RPL3* associated with reduced survival.^23^, ^24^ The first and most recent PD GWAS of a South American population was conducted in 2021, replicating an association at *SNCA* with 1497 participants.^25^

Recently, more multi‐ancestry studies have been conducted in PD, nominating novel loci for disease risk and age at onset including *ITGA8*, *SV2C*, and *WBSCR17* ^20^, ^26^, ^27^ The largest meta‐GWAS for PD, which included four ancestral populations, replicated 66 loci previously nominated in European studies as well as identified 12 novel loci: *MTF2*, *PIK3CA*, *ADD1*, *SYBU*, *IRS2*, *USP8*, *PIGL*, *FASN*, *MYLK2*, *USP25*, *EP300*, and *PPP6R2*.^7^

The largest non‐European PD GWAS was in East Asian populations; however, only a few novel loci have been nominated in that ancestry. Multi‐ancestry studies have nominated more novel variants in recent studies, but much more work is needed to better understand the risk for PD in non‐European populations. Only one locus discovered in a non‐European or multi‐ancestry study has been replicated (*ITGA8*).

**Amyotrophic Lateral Sclerosis**

**Largest European GWAS**: van Rheenen 2016

**Total samples**: 41,398

**Largest multi‐ancestry GWAS**: van Rheenen 2021

**Total samples**: 152,268

**Total non‐European samples**: 18,266

**% non‐European**: 12.00%

**Largest non‐European GWAS**: Wei 2019

**Ancestry**: EAS

**Total samples**: 4727

Loci discovered in non‐EUR or multi‐ancestry studies: 19

Loci replicated: 4 (*CAMK1G*, *CABIN1*/*SUSD2*, *GPX3*/*TNIP1*, *ACSL5*)

ALS GWAS in European populations have nominated a number of risk loci including *C9ORF72*, *UNC13A*, *C21orf2*, *SARM1*, *MOBP*, *SCFD1, TBKK1*, and *KIF5A*.^28^, ^29^

ALS is the only disease in our review for which more genome‐wide significant novel loci have been identified in a non‐European population than in the largest European‐only study. The first GWAS of individuals with Chinese Han ancestry identified *CAMK1G* and *CABIN1/SUSD2* as susceptibility loci for ALS.^30^ Later studies in the Han Chinese population nominated additional novel loci including *INPP5B, IQCF5/IQCF1, ITGA9, PFKP, MYO18B, ALCAM, OPCML*, *GPR133, TYW3/CRYZ*, and *FGD4*.^31^, ^32^ With a total of 12 genome‐wide significant loci, East Asian ancestry GWAS for ALS have nominated the most of any single non‐European population covered in our review.

Multi‐ancestry GWAS for ALS, which typically consist of European and East Asian ancestry populations, have been successful at nominating additional risk loci including *GPX3/TNIP1* and *ACSL5*.^29^, ^33^, ^34^ The largest ALS GWAS to date was a multi‐ancestry study including more than 150,000 individuals of European and East Asian ancestry. This study identified a total of 15 risk loci for ALS, replicating previously identified and nominating 5 novel loci: *SLC9A8/SPATA2, ERGIC1, NEK1, COG3*, and *PTPRN2*.^35^

Similar to PD, ALS GWAS including or focused on East Asian populations have made more progress than other non‐European populations for these diseases. However, much more work is still needed in all populations to progress potential precision medicine initiatives for ALS. Of the 19 loci discovered in non‐European or multi‐ancestry studies, only 4 have been replicated (*CAMK1G*, *CABIN1*/*SUSD2*, *GPX3*/*TNIP1*, *ACSL5*).

**Multiple Sclerosis**

**Largest European GWAS**: Patsopoulos 2019

**Total samples**: 115,803

**There are no multi‐ancestry studies in MS**.

**Largest non‐European GWAS**: Isobe 2015

**Ancestry**: AAC

**Total samples**: 2319

Loci discovered in non‐EUR or multi‐ancestry studies: 0

Loci replicated: 0

The largest GWAS meta‐analysis for MS included 115,803 individuals of European ancestry and found 82 significant genome‐wide associations with MS. This study was also the first to identify a risk locus for MS on chromosome X, and the identified genetic markers accounted for nearly 50% of the hereditary risk for MS.^36^

Studies in non‐European populations were more limited in MS than in the previous diseases discussed. The largest GWAS in African Americans was successful at replicating 21 of the loci previously identified in European populations but did not nominate any new risk loci at a genome‐wide significant level.^37^ The only other study nominated by our review process for MS in non‐European populations was conducted in a Mexican population. The study found four significant variants; however, these variants had limited regional support and the study was severely underpowered, with only 29 cases and 132 controls.^38^ Due to these limitations, we concluded that the variants identified in this study could not be classified as novel.

Sample sizes for MS GWAS are still relatively small, even for European populations. In addition, we did not find any multi‐ancestry studies through our search methods, highlighting a potential opportunity for further discovery for this disease.

**Frontotemporal Dementia**

**Largest European GWAS**: Ferrari 2014

**Total samples**: 12,928

**There are no multi‐ancestry or non‐European studies in FTD**.

Common risk loci nominated by previous European FTD studies include *C9ORF72, GRN*, and *MAPT*.^39^ The largest FTD GWAS in our review date range included ≈13,000 participants of European ancestry and nominated an additional locus in the *HLA‐DRA/HLA‐DRB5* region.^40^ This study was conducted in 2014, and although more recent GWAS of FTD have been performed, none have surpassed the sample size from the Ferrari study, and many have focused on smaller FTD subtypes.^41^, ^42^

No non‐European or multi‐ancestry GWAS were identified in our systematic review for FTD. Investigation of known genetic risk factors in non‐Europeans suggest that *C9ORF72* expansions may be quite rare in Chinese populations,^43^ highlighting the need for further research in this area.

**Myasthenia Gravis**

**Largest European GWAS**: Sakaue 2021

**Total samples**: 355,142

**Largest Multi‐ancestry GWAS**: Sakaue 2021

**Total samples**: 533,853

**Total non‐European samples**: 178,711

**% non‐European**: 33.47%

**Largest non‐European GWAS**: Na 2014

**Ancestry**: EAS

**Total samples**: 259

Loci discovered in non‐EUR or multi‐ancestry studies: 0

Loci replicated: 0

Known loci for MG include *PTPN22*, *CTLA4*, *HLA‐DQA1, ZBTB10*, and *TNFRSF11A*,^44^, ^45^ all nominated in European‐based GWAS. The most recent GWAS for MG nominated additional loci at *CHRNA1, SFMBT2*, and *FAM76B*, although the latter two did not replicate.^46^ The largest European and multi‐ancestry GWAS for MG to date were both performed in the same study, leveraging 533,853 total samples from Japanese, UK, and Finnish‐based biobanks. However, with only 278 cases, the effective sample size (4/(1/ncase+1/ncontrol)) for the meta‐analysis was insufficiently powered and no new loci were nominated for MG.^47^

In non‐European populations, the literature review identified one Korean GWAS for MG. However, this study was small and did not identify any loci meeting genome‐wide significance.^48^ Other studies have found that there is earlier onset of MG in Asian populations, and higher prevalence of the ocular form in Asian children, highlighting the importance of continued discovery efforts for MG in non‐European populations.^49^

**Lewy Body Dementia**

**Largest European GWAS**: Chia 2021

**Total samples**: 7372

**There are no multi‐ancestry or non‐European studies in LBD**.

Previously nominated risk loci for LBD include *GBA*, *APOE*, and *SNCA*.^50^, ^51^ LBD can be hard to diagnose as there are a number of clinical and genetic overlaps with AD and PD, which may be one of the reasons why there is still limited genetic research for LBD in both European and non‐European populations.^50^, ^51^, ^52^

We found no LBD GWAS in any single non‐European ancestry populations or any multi‐ancestry studies through our search methods. Concrete data on the prevalence of LBD in diverse ancestries is difficult to acquire, showing a potential opportunity for valuable future research.

**Vascular dementia**

**Largest European GWAS**: Moreno‐Grau 2019

**Total samples**: 4830

**Largest Multi‐ancestry GWAS**: Fongang 2022

**Total samples**: 482,088

**Total non‐European samples**: 11,590

**% non‐European**: 2.40%

**There are no non‐European studies in VaD**.

Despite an approximated prevalence of about 15%–20% in all dementia cases,^53^ vascular dementia (or VaD) remains difficult to study because of the uncertainty of diagnosis. In fact, only two studies on VaD passed our criteria and only one of these found genome‐wide significant novel loci. The first study was a European GWAS that looked at vascular, mixed, and pure AD phenotypes and nominated loci at *ANKRD31* and *NDUFAF6*.^54^ The second study that passed our criteria was a multi‐ancestry GWAS for all‐cause and VaD including participants from European, African, Asian, and Hispanic ancestries, but did not find any significant novel loci.^55^

VaD prevalence and risk appears to be higher in South Asian ancestries compared to European or Chinese populations.^56^, ^57^ Additional studies have suggested that African Americans are most likely to be admitted to inpatient care with a VaD primary diagnosis.^58^ Despite these findings, there are still limited genetic studies for VaD, and we found no single non‐European GWAS, highlighting the need for future research.

## 4 DISCUSSION

This review highlights the lack of ancestral diversity in genetic research across NDD GWAS over the past decade. Current research suggests that including non‐European populations can improve our understanding of the genetic architecture of disease through novel ancestry‐specific discoveries, increased statistical power awarded by studying diverse haplotype structures, and the identification of loci with heterogeneous effects across populations.^59^ Although we identified 50 novel loci discovered in diverse populations in our review, only six of the loci have been replicated, highlighting the need for larger numbers of non‐Europeans to be included in genetic studies.

Furthermore, discrepancies in how diseases are diagnosed across populations introduce additional heterogeneity, complicating the replication process. Variations in diagnostic criteria, health care access, and cultural factors can lead to differences in disease classification and severity, influencing the observed genetic associations. For instance, in NDDs, diagnostic criteria may vary between populations due to differences in symptom presentation, age at onset, or cultural perceptions of cognitive decline. These differences not only affect the phenotypic characterization of the disease but also may impact the genetic underpinnings identified through GWAS. Consequently, the failure to replicate GWAS findings across populations may stem not only from genetic diversity or insufficient power in smaller studies but also from variations in disease definitions and diagnostic practices. Addressing these challenges necessitates not only larger and more diverse sample sizes but also efforts to harmonize diagnostic criteria and standardize phenotypic assessments across populations.

In addition, although we looked at seven genetic ancestry groups, these “buckets” do not capture the true diversity of global populations. The African continent is known to have high genetic diversity, yet individuals of African ancestry are routinely grouped into a single category.^60^ In fact, we found no studies investigating South Asian (SAS) or continental African (AFR) populations. After investigating the cohorts in our review, we noted that although there were multiple “African” labeled studies, none of them directly investigated individuals in continental Africa, instead looking at African American or other African admixed populations. It is critical to mention the reference population used to define the specific population, to prevent the misattribution of genetic features across ancestries. Grouping all participants with any African ancestry into a generalized African category obscures the significant issue of inadequate representation of continental Africans.

In addition, there has been very little research done on admixed populations, and how the combinations of different ancestries affect SNP frequencies and/or gene expression. A GWAS in a Caribbean Hispanic admixed population found that the frequency of a novel locus spanning *FBXL7* varied greatly, from 1% in those with European ancestry to 20% in African Americans.^13^

Furthermore, previous research has shown that the transferability of polygenic risk scores from African Americans to various African populations is highly unreliable.^61^ The substantial genetic and environmental disparities among individuals of African descent underscore the urgent need to improve diversity in genetic studies.

Our review is not without limitations. One limitation is that due to studies using different versions of summary statistics, different release versions of cohorts, and different filtering and quality control pipelines, we could not accurately calculate the percentage of sample overlap across studies. It is important to note that disease cohorts or summary statistics are often included in multiple GWAS. This is especially common in multi‐ancestry meta‐analyses, which often rely on publicly available summary statistics to increase power.^7^ Potentially high sample overlap between studies such as Wightman et al. and Schwartzentruber et al., which both include cohorts like the UK Biobank, may spark debate about whether they can be counted as completely “unique” GWAS. However, we chose to include any GWAS in our review despite potential overlap if they included any additional or varying cohorts to paint an overall picture of the state of diversity in GWAS. The number of unique participants included in each subsequent GWAS across ancestries would be an interesting comparison for a future study, although it will likely highlight very little overlap in diverse ancestry studies as there are still too few studies being conducted in those populations.

Another limitation to our review is that we chose to focus on a 10‐year time period. This time‐span was chosen to cover the trends in the most recent decade in GWAS research in‐depth. However, many older GWAS, including some in diverse ancestry populations, were conducted prior to 2012. In addition, important diverse ancestry studies have since been published beyond our search date of the National Institutes of Health (NIH) Library of Medicine and the GWAS Catalog. We chose to highlight a few of these foundational studies in our review despite being outside of the designated 10‐year period. Future studies may choose a wider time span for reviewing GWAS studies, which may highlight an even larger disparity between European and diverse ancestry studies prior to 2012.

### 4.1 Diversity in SNP discovery

Although six of the eight NDDs we investigated had non‐European representation, only PD had >1000 cases and >30% non‐European samples (Table 1). Furthermore, no new significant loci have been identified in diverse population studies for MS, LBD, FTD, VaD, or MG. Of the 50 novel significant loci that have been discovered, only six have been replicated. Although the largest non‐European cohort in MG included almost 180,000 samples, only 81 MG cases were included. A GWAS with fewer than 1000 cases is unlikely to achieve sufficient statistical power for SNP discovery in polygenic diseases where multiple loci with small effect sizes are generally expected.^62^, ^63^, ^64^ Alzheimer's, the most well‐funded of all the NDDs, has less than 3% diversity among cases in genetic studies. The incorporation of studies that lack statistical power and replicability minimizes the true imbalance between European and non‐European studies, maintaining a Eurocentric bias.

Furthermore, the variation in minor allele frequency (MAF) thresholds across studies, as detailed in Table 1, illustrates a methodological challenge in such genetic research. The ability to detect rarer genetic variants in smaller studies is often limited by higher MAF thresholds, potentially omitting crucial findings in these populations. This limitation is particularly significant, as it suggests that some variants currently identified as present only in European populations may, in fact, be found in other ancestries as more comprehensive and diverse samples become available. The current perception of ancestry‐specific genetic markers may thus evolve with the inclusion of broader, more representative data sets. Such developments could lead to the identification of previously unrecognized genetic diversity and the re‐evaluation of the geographical and ancestral specificity of certain genetic variants.

In addition, many genetic association studies in East Asian populations did not meet our review criteria because they were not conducted on a genome‐wide scale. Instead, these studies often investigated only one or a small group of SNPs that had been previously associated with disease in European populations, potentially missing associations that are specific to non‐European populations.

In addition, our study may not have captured some non‐European research, as we excluded studies not published in English. This decision was based on the understanding that over 95% of scientific literature is published in English; however, we acknowledge that this represents a slight limitation of our work.^65^

Many loci identified in European populations have heterogeneous effects or ancestry‐specific SNP associations. For example, although the *APOE* alleles account for around a quarter of overall heritability for AD in Europeans,^8^, ^16^ several studies suggest that the *APOE4* allele has a weaker effect in African ancestry^66^, ^67^ and Caribbean Hispanic^68^ populations. The effect has been found to be greater in Japanese populations.^66^, ^67^ Heterogeneity of risk at *APOE* ε4 has been quantified in a recent multi‐ancestry meta‐analysis, with an estimated variability in effect size (I2) across ancestries of up to 85%. Fifty percent or more of that risk heterogeneity was found to be attributable to genetic ancestry differences, suggesting that although *APOE* is still an important locus for AD risk across populations, the strength of the effect of *APOE* on AD risk varies with genetic ancestral background.^6^ We believe that examination of local ancestry at loci with such global differences may help discern whether locus‐specific inheritance patterns modulate disease risk.

Similarly, *C9ORF72* is one of the most common risk factors for ALS. However, the frequency of the *C9ORF72* expansion is lower in Chinese populations (0.3%) as compared to European populations (7%).^29^ Recent research suggests that commonly used genetic tests to diagnose ALS may be less accurate in non‐European ancestry patients because they are less likely to carry the *C9ORF72* structural variant.^69^

Some SNPs with large effect sizes do not exist or are extremely rare in certain ancestry groups. Variants in *ABCA7*, for example, increase AD risk more in individuals of African ancestry than in those of European ancestry.^70^ In fact, *ABCA7* has a comparable effect size to *APOE* in individuals of African ancestry.^10^ Genetic variants in *LRRK2, GBA*, and *SNCA*, which have been associated with increased risk of PD in European ancestry populations, appear to have a negligible effect in individuals from India.^71^, ^72^, ^73^, ^74^ Without studying diverse populations, researchers would miss the population‐specific effects of these loci and potential therapeutic targets that modify their effects.

### 4.2 Looking forward

Despite the inequalities highlighted above, progress is being made. Researchers in AD are taking a strong multi‐modal approach to increasing diversity. The Multi‐Partner Consortium to Expand Dementia Research in Latin America (ReDLat) is leveraging “on the ground” connections with research communities in Latin America and the Caribbean to grow a diverse database of dementia resources.^75^, ^76^ The Alzheimer's Disease NeuroImaging (ADNI) study is growing more inclusive cell lines and generating partner data for multiple ancestrally diverse samples.^77^ The NIH's Center for Alzheimer's and Related Dementias (CARD) is filling diversity gaps by creating training materials, generating data to complement existing efforts, and providing open science support for researchers in diverse communities.

Multiple efforts are also underway in the PD space. The Genetic Architecture of Parkinson disease in India (GAP‐India) plans to develop a large clinical/genomic biobank in India.^71^ The Latin American Research Consortium on the Genetics of PD (LARGE‐PD) aims to address inclusivity and genomic differences within and across Latino populations. Finally, the Global Parkinson's Genetics Program (GP2) aims to genotype >150,000 individuals from around the world. GP2‐funded projects include the Black and African Americans Connections to Parkinson's Disease Study (BLAACPD), which seeks to assess the genetic architecture of Black and African American individuals with PD, as well as healthy subjects, from across the United States.^78^ GP2 is motivated to increase diversity not only just among samples recruited into studies, but also in the investigators making use of the data, providing training and resources to ensure that all researchers are on an open and equal field of play.^79^ A list of ongoing efforts for increasing diversity in NDD genetic research, including atypical dementias, can be found in Table S3.

**FIGURE 2:**
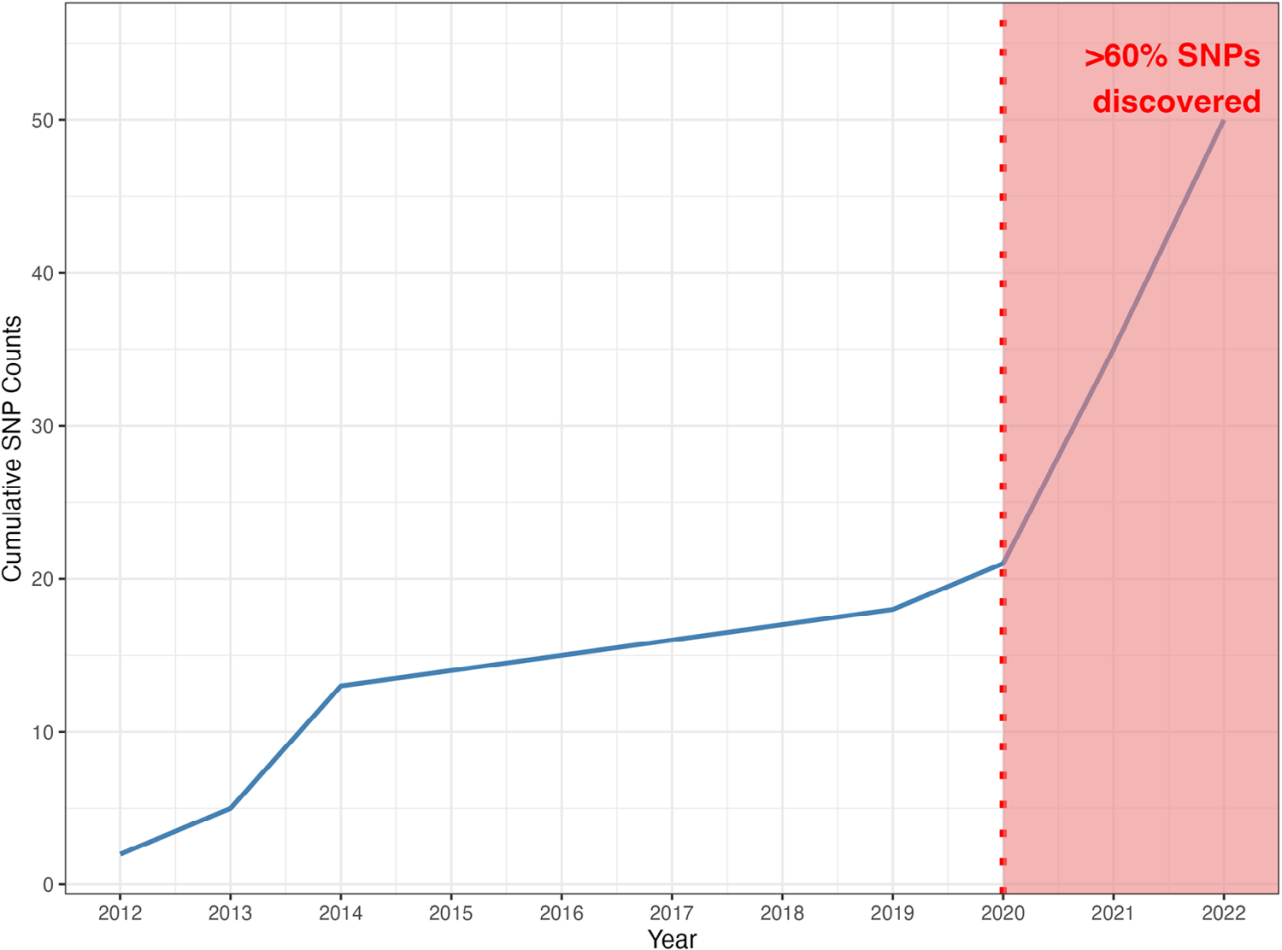
Cumulative count of discovered SNPs from 2012 through 2022. Notably, more than 60% of the SNPs were identified in the period between 2020 through 2022. SNPs, single nucleotide polymorphisms.

These efforts are paying off. More than 60% of the NDD‐associated loci discovered in non‐European or multi‐ancestry populations were identified in the period between 2020 and 2022 (Figure 2). Over the past 10 years, there has been a steady increase in the proportion of non‐European samples included in genetic studies (Figure 1B). With the increase in diverse samples in recent years, there has also been a growing interest in the use of multi‐ancestry analyses to discover, fine‐map, and assess heterogeneity at disease risk loci, particularly in AD, PD, and ALS (Figure 1A).

In fact, we are already seeing the benefits of increased diversity on genetic discovery in NDD research. A 2023 GWAS using African and African American samples collected by GP2 and 23andMe, and co‐lead by researchers in Nigeria and NIH, found a novel *GBA1* locus that is rare in other populations.^80^ We anticipate that in the future, leveraging multiple ancestries will continue to improve fine‐mapping resolution to prioritize causal variants,^5^ increase access to and reduce bias in precision medicine practices such as polygenic risk prediction,^1^ and drive many new discoveries in the genetics of NDDs.

## 5 CONCLUSION

Our systematic review highlights a striking disparity in the representation of diverse genetic ancestry populations in NDD research. We also show the variability among the novel loci, noting that no locus found in a single non‐European population has been replicated in another population, emphasizing the urgent need for greater inclusivity to advance our understanding of these complex conditions and develop more equitable precision medicine approaches. Efforts to bridge this gap and promote diversity in genetic studies are vital for achieving meaningful progress in the diagnosis, treatment, and prevention of NDDs across global populations.

## CONFLICT OF INTEREST STATEMENT

C.J, K.S.L., L.J., H.L.L., and M.A.N.’s participation in this project was part of a competitive contract awarded to DataTecnica LLC by the National Institutes of Health to support open science research. M.A.N. also currently serves on the scientific advisory board for Character Bio Inc. and is a scientific founder at Neuron23 Inc. J.S.Y. serves on the scientific advisory board for the Epstein Family Alzheimer's Research Collaboration. J.L., L.H., D.P., J.K., S.B., N.T., I.M., C.B., and A.B.S. have no conflicts of interest. Author disclosures are available in the Supporting Information.

## Supporting information

Supporting Information

Supporting Information

Supporting Information

Supporting Information

Supporting Information
