# Supplementary material for: Assessing the lack of diversity in genetics research across neurodegenerative diseases: A systematic review of the GWAS Catalog and literature
Source: Alzheimers Dement. 2024 Jun 21;20(8):5740–56. doi: 10.1002/alz.13873 (PMC11350004; doi:10.1002/alz.13873)

**Supplemental Figure 1: PRISMA flow diagram for systematic review of published GWAS studies and pubmed literature review from NLM.** N in the figure relates to the number of published studies as of April 28th, 2022.

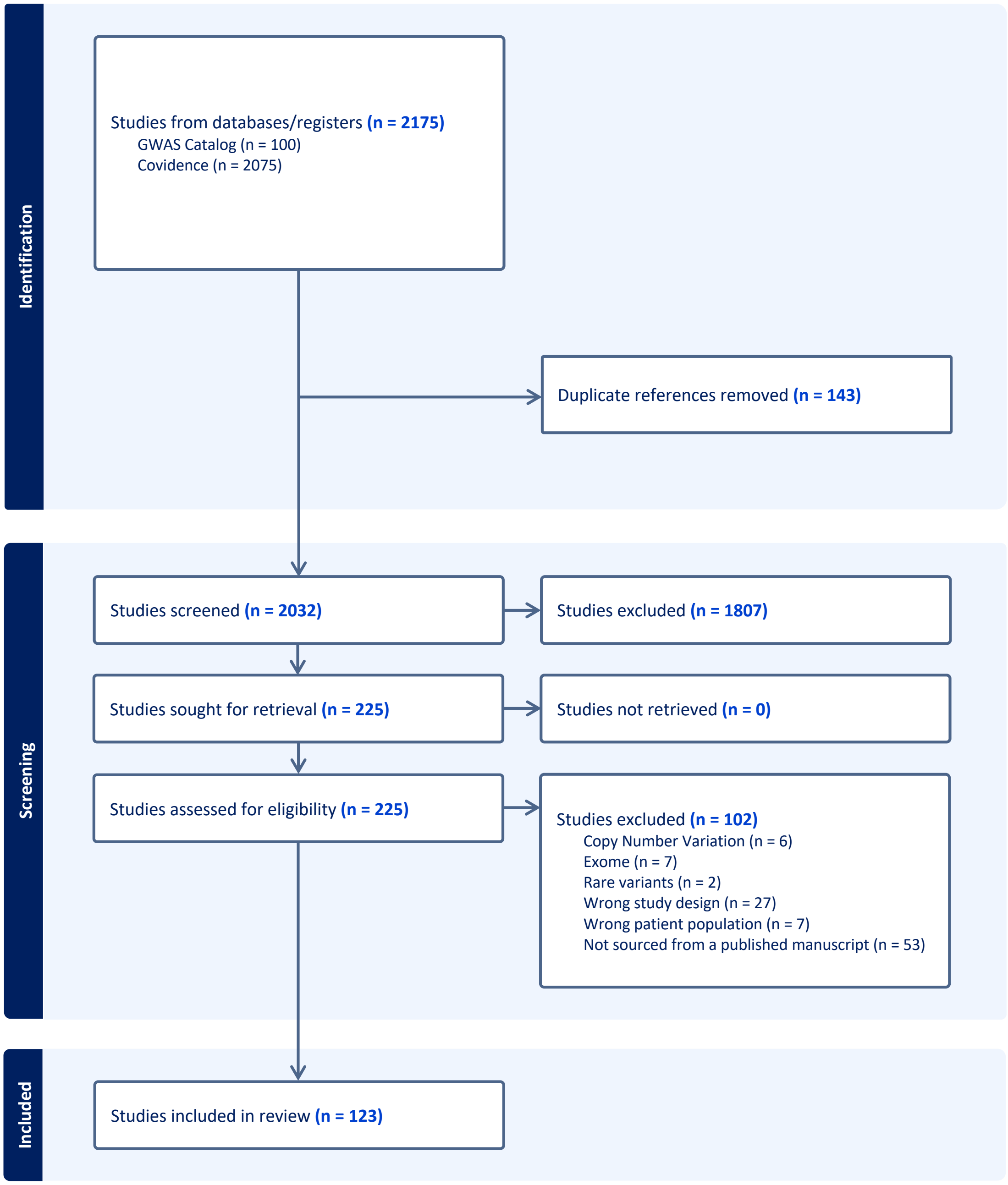

Supplement: Supplementary file 1 — Supporting Information [file ALZ-20-5740-s004.pdf]
