# Supplementary material for: Assessing the lack of diversity in genetics research across neurodegenerative diseases: A systematic review of the GWAS Catalog and literature
Source: Alzheimers Dement. 2024 Jun 21;20(8):5740–56. doi: 10.1002/alz.13873 (PMC11350004; doi:10.1002/alz.13873)
Supplement: Supplementary file 2 — Supporting Information [file ALZ-20-5740-s002.docx]

**Supplementary Table 1: Search terms used in our systematic review.**  We searched both the GWAS Catalog and also did a search with the National Library of Medicine.

| **Search Location** | **Search Terms** |
| --- | --- |
| GWAS Catalog | Alzheimer’s disease; late-onset Alzheimer's disease; family history of Alzheimer’s disease; multiple sclerosis; dementia; frontotemporal dementia; lewy body dementia; vascular dementia; amyotrophic lateral sclerosis; sporadic amyotrophic lateral sclerosis; Parkinson’s disease; young adult-onset Parkinsonism; myasthenia gravis and late-onset myasthenia gravis. |
| National Library of Medicine search #1: | Alzheimer disease[mh] OR Parkinson disease[mh] OR Lewy Body Disease[mh] OR Amyotrophic Lateral Sclerosis[mh] OR Frontotemporal Dementia[mh] OR Multiple Sclerosis[mh] OR Dementia, Vascular[mh] OR Myasthenia Gravis[mh] OR Alzheimer[tiab] OR alzheimer's[tiab] OR "Alzheimer-type dementia*"[tiab] OR "parkinson's disease"[tiab] OR "parkinson disease"[tiab] OR "lewy body disease"[tiab] OR "lewy body dementia"[tiab] OR "frontotemporal dementia"[tiab] OR "frontotemporal lobe dementia"[tiab] OR "amyotrophic lateral sclerosis"[tiab] OR "Lou Gehrig Disease"[tiab] OR "Lou-Gehrig's Disease"[tiab] OR "Lou-Gehrigs Disease"[tiab] OR "multiple sclerosis"[tiab] OR "vascular dementia"[tiab] OR "myasthenia gravis"[tiab] |
| National Library of Medicine search #2: | Genome-Wide Association Study[mh] OR "genome-wide association stud*"[tiab] OR "genome wide association stud*"[tiab] OR "whole genome association stud*"[tiab] OR "genome wide association analysis"[tiab] OR "genome wide association scan"[tiab] OR "genome wide-association analysis"[tiab] OR "genome-wide association scan"[tiab] OR genome-wide[tiab] OR "genome wide"[tiab] OR GWAS[tiab] |
| National Library of Medicine search #3: | African Continental Ancestry Group[mh] OR Continental Population Groups[mh] OR Black People[mh] OR Blacks[mh] OR Black OR African American[mh] OR Asian Continental Ancestry Group[mh] OR Asian People[mh] OR East Asian People[mh] OR Central Asian People[mh] OR North Asian People[mh] OR Southeast Asian People[mh] OR West Asian People[mh] OR Asians[mh] OR Caribbean People[mh] OR Middle Eastern People[mh] OR Central American People[mh] OR European People[mh] OR Middle Eastern and North Africans[mh] OR North American People[mh] OR Oceanians[mh] OR Oceanic Ancestry Group[mh] OR Native Hawaiian OR Other Pacific Islander[mh] OR South American People[mh] OR Indians, South American[mh] OR European Continental Ancestry Group[mh] OR white people[mh] OR whites[mh] OR ethnicity[mh] OR racial groups[mh] OR "central american*"[tiab] OR "south american*"[tiab] OR "north american*"[tiab] OR european*[tiab] OR "oceanic ancestry"[tiab] OR "european continental ancestry"[tiab] OR "middle eastern"[tiab] OR "north african*"[tiab] OR African*[tiab] OR Asian*[tiab] OR Korean*[tiab] OR Chinese*[tiab] OR Japanese[tiab] OR Indian[tiab] OR Hispanic*[tiab] OR Latin*[tiab] OR Mexican*[tiab] OR Colombian[tiab] OR Arab[tiab] OR Iranian[tiab] OR Caucasian[tiab] OR "Alaska native"[tiab] OR "native Hawaiian*" OR admixed[tiab] OR ancestry[tiab] OR multi-ancestry[tiab] OR multiancestry[tiab] OR transethnic[tiab] OR trans-ethnic[tiab] OR multi-ethnic*[tiab] |
