# Supplementary material for: Assessing the lack of diversity in genetics research across neurodegenerative diseases: A systematic review of the GWAS Catalog and literature
Source: Alzheimers Dement. 2024 Jun 21;20(8):5740–56. doi: 10.1002/alz.13873 (PMC11350004; doi:10.1002/alz.13873)
Supplement: Supplementary file 3 — Supporting Information [file ALZ-20-5740-s001.docx]

**Supplementary Table 2: All studies passing our filters.** This table includes all 123 studies that passed our inclusion criteria and review.

| **Study ID** | **Year** | **Title** | **Reviewer Name** | **Author** | **Disease** | **Ancestry** |
| --- | --- | --- | --- | --- | --- | --- |
| Ahmeti 2013 | 2013 | Age of onset of amyotrophic lateral sclerosis is modulated by a locus on 1p34.1 | Consensus | Ahmeti K. | ALS | EUR |
| Andlauer 2016 | 2016 | Novel multiple sclerosis susceptibility loci implicated in epigenetic regulation | Consensus | Andlauer T. | MS | EUR |
| Bandres-Ciga 2016 | 2016 | Genome-wide assessment of Parkinson's disease in a Southern Spanish population | Consensus | Bandres-Ciga S. | PD | EUR |
| Bandres-Ciga 2019 | 2019 | The Genetic Architecture of Parkinson Disease in Spain: Characterizing Population-Specific Risk, Differential Haplotype Structures, and Providing Etiologic Insight | Consensus | Bandres-Ciga S. | PD | EUR |
| Beecham 2013 | 2013 | Analysis of immune-related loci identifies 48 new susceptibility variants for multiple sclerosis | Consensus | International Multiple Sclerosis Genetics Consortium (IMSGC) | MS | EUR |
| Beecham 2014 | 2014 | Genome-wide association meta-analysis of neuropathologic features of Alzheimer's disease and related dementias | Consensus | Beecham G. | LBD,AD | EUR |
| Beecham 2015 | 2015 | PARK10 is a major locus for sporadic neuropathologically confirmed Parkinson disease | Consensus | Beecham G. | PD | EUR |
| Bellenguez 2022 | 2022 | New insights into the genetic etiology of Alzheimer's disease and related dementias | Consensus | Bellenguez C. | AD | EUR |
| Benyamin 2017 | 2017 | Cross-ethnic meta-analysis identifies association of the GPX3-TNIP1 locus with amyotrophic lateral sclerosis | Consensus | Benyamin B. | ALS | EAS, EUR |
| Bi 2020 | 2020 | A Fast and Accurate Method for Genome-Wide Time-to-Event Data Analysis and Its Application to UK Biobank | Consensus | Bi W. | PD,AD | EUR |
| Blauwendraat 2019 | 2019 | Parkinson's disease age at onset genome-wide association study: Defining heritability, genetic loci, and α-synuclein mechanisms | Consensus | Blauwendraat C. | PD | EUR |
| Blauwendraat 2020 | 2020 | Genetic modifiers of risk and age at onset in GBA associated Parkinson's disease and Lewy body dementia | Consensus | Blauwendraat C. | LBD,PD | EUR |
| Blauwendraat 2021 | 2021 | Investigation of Autosomal Genetic Sex Differences in Parkinson's Disease | Consensus | Blauwendraat C. | PD | EUR |
| Bracher-Smith 2022 | 2022 | Whole genome analysis in APOE4 homozygotes identifies the DAB1-RELN pathway in Alzheimer's disease pathogenesis | Consensus | Bracher-Smith M. | AD | EUR |
| Brolin 2022 | 2022 | Insights on Genetic and Environmental Factors in Parkinson's Disease from a Regional Swedish Case-Control Cohort | Consensus | Brolin K. | PD | EUR |
| Chang 2017 | 2017 | A meta-analysis of genome-wide association studies identifies 17 new Parkinson's disease risk loci | Consensus | Chang D. | PD | EUR |
| Chen 2016 | 2016 | A genome-wide association study on amyotrophic lateral sclerosis in the Taiwanese Han population | Consensus | Chen C. | ALS | EAS |
| Chia 2021 | 2021 | Genome sequencing analysis identifies new loci associated with Lewy body dementia and provides insights into its genetic architecture | Consensus | Chia R. | LBD | EUR |
| Chia 2022 | 2022 | Identification of genetic risk loci and prioritization of genes and pathways for myasthenia gravis: a genome-wide association study | Consensus | Chia R. | MG | EUR |
| Chung 2012 | 2012 | Genomic determinants of motor and cognitive outcomes in Parkinson's disease | Consensus | Chung S. | PD | EUR |
| Chung 2022 | 2022 | Genome-wide association and multi-omics studies identify MGMT as a novel risk gene for Alzheimer's disease among women | Consensus | Chung J. | AD | EUR |
| Cummings 2012 | 2012 | Genome-Wide Association and Linkage Study in the Amish Detects a Novel Candidate Late-Onset Alzheimer Disease Gene | Consensus | Cummings A. | AD | EUR |
| Davis 2013 | 2013 | Parkinson disease loci in the mid-western Amish | Consensus | Davis M. | PD | EUR |
| Deng 2013 | 2013 | Genome-wide association analyses in Han Chinese identify two new susceptibility loci for amyotrophic lateral sclerosis | Consensus | Deng M. | ALS | EAS |
| Diekstra 2014 | 2014 | C9orf72 and UNC13A are shared risk loci for amyotrophic lateral sclerosis and frontotemporal dementia: a genome-wide meta-analysis | Consensus | Diekstra F. | ALS,FTD | EUR |
| Ferrari 2014 | 2014 | Frontotemporal dementia and its subtypes: a genome-wide association study | Consensus | Ferrari R. | FTD | EUR |
| Ferrari 2015 | 2015 | A genome-wide screening and SNPs-to-genes approach to identify novel genetic risk factors associated with frontotemporal dementia | Consensus | Ferrari R. | FTD | EUR |
| Ferrari 2017 | 2017 | Effects of Multiple Genetic Loci on Age at Onset in Frontotemporal Dementia | Consensus | Ferrari R. | FTD | EUR |
| Fogh 2014 | 2014 | A genome-wide association meta-analysis identifies a novel locus at 17q11.2 associated with sporadic amyotrophic lateral sclerosis | Consensus | Fogh I. | ALS | EUR |
| Fogh 2016 | 2016 | Association of a Locus in the CAMTA1 Gene With Survival in Patients With Sporadic Amyotrophic Lateral Sclerosis | Consensus | Fogh I. | ALS | EUR |
| Fongang 2022 | 2022 | A meta-analysis of genome-wide association studies identifies new genetic loci associated with all-cause and vascular dementia | Consensus | Fongang B. | VaD | AAC,EUR,  AMR,EAS |
| Foo 2017 | 2017 | Genome-wide association study of Parkinson's disease in East Asians | Consensus | Foo J. | PD | EAS |
| Foo 2020 | 2020 | Identification of Risk Loci for Parkinson Disease in Asians and Comparison of Risk Between Asians and Europeans: A Genome-Wide Association Study | Consensus | Foo J. | PD | EAS,EUR |
| Gaj 2012 | 2012 | Identification of a late onset Alzheimer's disease candidate risk variant at 9q21.33 in Polish patients | Consensus | Gaj P. | AD | EUR |
| Gan-Or 2015 | 2015 | The Alzheimer disease BIN1 locus as a modifier of GBA-associated Parkinson disease | Consensus | Gan-Or Z. | PD | EUR |
| Giacalone 2015 | 2015 | Analysis of genes, pathways and networks involved in disease severity and age at onset in primary-progressive multiple sclerosis | Consensus | Giacalone G. | MS | EUR |
| Goris 2014 | 2014 | No evidence for shared genetic basis of common variants in multiple sclerosis and amyotrophic lateral sclerosis | Consensus | Goris A. | ALS,MS | EUR |
| Gregersen 2012 | 2012 | Risk for Myasthenia Gravis Maps to a (151)Pro -> Ala Change in TNIP1 and to Human Leukocyte Antigen-B*08 | Consensus | Gregersen P. | MG | EUR |
| Grover 2022 | 2022 | Genome-wide Association and Meta-analysis of Age at Onset in Parkinson Disease: Evidence From the COURAGE-PD Consortium | Consensus | Grover S. | PD | EAS,EUR |
| Guerreiro 2018 | 2018 | Investigating the genetic architecture of dementia with Lewy bodies: a two-stage genome-wide association study | Consensus | Guerreiro R. | LBD | EUR |
| Herold 2016 | 2016 | Family-based association analyses of imputed genotypes reveal genome-wide significant association of Alzheimer's disease with OSBPL6, PTPRG, and PDCL3 | Consensus | Herold C. | AD | EUR |
| Hill-Burns 2014 | 2014 | Identification of a novel Parkinson's disease locus via stratified genome-wide association study | Consensus | Hill-Burns E. | PD | EUR |
| Hill-Burns 2016 | 2016 | Identification of genetic modifiers of age-at-onset for familial Parkinson's disease | Consensus | Hill-Burns E. | PD | EUR |
| Hirano 2015 | 2015 | A genome-wide association study of late-onset Alzheimer's disease in a Japanese population | Consensus | Hirano A. | AD | EAS |
| Horimoto 2021 | 2021 | Admixture mapping reveals the association between Native American ancestry at 3q13.11 and reduced risk of Alzheimer's disease in Caribbean Hispanics | Consensus | Horimoto A. | AD | AAC |
| Hu 2016 | 2016 | A Pooling Genome-Wide Association Study Combining a Pathway Analysis for Typical Sporadic Parkinson's Disease in the Han Population of Chinese Mainland | Consensus | Hu Y. | PD | EAS |
| Huang 2017 | 2017 | A common haplotype lowers PU.1 expression in myeloid cells and delays onset of Alzheimer's disease | Consensus | Huang K. | AD | EUR |
| Iacoangeli 2020 | 2020 | Genome-wide Meta-analysis Finds the ACSL5-ZDHHC6 Locus Is Associated with ALS and Links Weight Loss to the Disease Genetics | Consensus | Iacoangeli A. | ALS | EAS, EUR |
| Isobe 2015 | 2015 | An ImmunoChip study of multiple sclerosis risk in African Americans | Consensus | Isobe N. | MS | AAC |
| Jansen 2019 | 2019 | Genome-wide meta-analysis identifies new loci and functional pathways influencing Alzheimer's disease risk | Consensus | Jansen I. | AD | EUR |
| Jia 2021 | 2021 | Prediction of Alzheimer's disease using multi-variants from a Chinese genome-wide association study | Consensus | Jia L. | AD | EAS |
| Jokubaitis 2022 | 2022 | Not all roads lead to the immune system: the genetic basis of multiple sclerosis severity | Consensus | Jokubaitis V. | MS | EUR |
| Jonsson 2013 | 2013 | Variant of TREM2 associated with the risk of Alzheimer's disease | Consensus | Jonsson T. | AD | EUR |
| Jun-G 2016 | 2016 | A novel Alzheimer disease locus located near the gene encoding tau protein | Consensus | Jun G. | AD | EUR |
| Jun-G 2017 | 2017 | Transethnic genome-wide scan identifies novel Alzheimer's disease loci | Consensus | Jun G. | AD | EAS, MDE,  AAC, EUR |
| Kamboh 2012 | 2012 | Genome-wide association analysis of age-at-onset in Alzheimer's disease | Consensus | Kamboh M. | AD | EUR |
| Kamboh 2012 | 2012 | Genome-wide association study of Alzheimer's disease | Consensus | Kamboh M. | AD | EUR |
| Kang 2021 | 2021 | Potential Novel Genes for Late-Onset Alzheimer's Disease in East-Asian Descent Identified by APOE-Stratified Genome-Wide Association Study | Consensus | Kang S. | AD | EAS |
| Kim 2022 | 2022 | Multi-ancestry genome-wide meta-analysis in Parkinson's disease | Consensus | Kim J. | PD | AMR, AAC,  EAS, EUR |
| Kulminski 2018 | 2018 | Strong impact of natural-selection-free heterogeneity in genetics of age-related phenotypes | Consensus | Kulminski A. | AD | EUR |
| Kunkle 2019 | 2019 | Genetic meta-analysis of diagnosed Alzheimer's disease identifies new risk loci and implicates AB, tau, immunity and lipid processing | Consensus | Kunkle B. | AD | EUR |
| Kunkle 2021 | 2021 | Novel Alzheimer Disease Risk Loci and Pathways in African American Individuals Using the African Genome Resources Panel: A Meta-analysis | Consensus | Kunkle B. | AD | AAC |
| Kwee 2012 | 2012 | A high-density genome-wide association screen of sporadic ALS in US veterans | Consensus | Kwee L. | ALS | EUR |
| Lake 2022 | 2022 | Multi-ancestry meta-analysis and fine-mapping in Alzheimer's Disease | Consensus | Lake J. | AD | EAS, AAC,  EUR |
| Lambert 2013 | 2013 | Meta-analysis of 74,046 individuals identifies 11 new susceptibility loci for Alzheimer's disease | Consensus | Lambert J. | AD | EUR |
| Lee 2017 | 2017 | Single-nucleotide polymorphisms are associated with cognitive decline at Alzheimer's disease conversion within mild cognitive impairment patients | Consensus | Lee E. | AD | EUR |
| Li 2021 | 2021 | Use of Deep-Learning Genomics to Discriminate Healthy Individuals from Those with Alzheimer's Disease or Mild Cognitive Impairment | Consensus | Li L. | AD | EUR |
| Li 2021 | 2021 | Genetic Modifiers of Age at Onset for Parkinson's Disease in Asians: A Genome-Wide Association Study | Consensus | Li C. | PD | EAS, EUR |
| Li 2022 | 2022 | Genetic Determinants of Survival in Parkinson's Disease in the Asian Population | Consensus | Li C. | PD | EAS |
| Lill 2012 | 2012 | Comprehensive research synopsis and systematic meta-analyses in Parkinson's disease genetics: The PDGene database | Consensus | Lill C. | PD | EAS, EUR |
| Lo 2019 | 2019 | Identification of genetic heterogeneity of Alzheimer's disease across age | Consensus | Lo M. | AD | EUR |
| Loesch 2021 | 2021 | Characterizing the Genetic Architecture of Parkinson's Disease in Latinos | Consensus | Loesch D. | PD | AMR |
| Marioni 2018 | 2018 | GWAS on family history of Alzheimer's disease | Consensus | Marioni R. | AD | EUR |
| Martinelli-Boneschi 2012 | 2012 | A genome-wide association study in progressive multiple sclerosis | Consensus | Martinelli-Boneschi F. | MS | EUR |
| Matesanz 2012 | 2012 | Genome-wide association study of multiple sclerosis confirms a novel locus at 5p13.1 | Consensus | Matesanz F. | MS | EUR |
| McLaughlin 2015 | 2015 | A second-generation Irish genome-wide association study for amyotrophic lateral sclerosis | Consensus | McLaughlin R. | ALS | EUR |
| Mez 2017 | 2017 | Two novel loci, COBL and SLC10A2, for Alzheimer's disease in African Americans | Consensus | Mez J. | AD | AAC |
| Miron 2018 | 2018 | CDK5RAP2 gene and tau pathophysiology in late-onset sporadic Alzheimer's disease | Consensus | Miron J. | AD | EUR |
| Miyashita 2013 | 2013 | SORL1 is genetically associated with late-onset Alzheimer's disease in Japanese, Koreans and Caucasians | Consensus | Miyashita A. | AD | EAS,EUR |
| Moreno-Grau 2019 | 2019 | Genome-wide association analysis of dementia and its clinical endophenotypes reveal novel loci associated with Alzheimer's disease and three causality networks: The GR@ACE project | Consensus | Moreno-Grau S. | VaD,AD | EUR |
| Na 2014 | 2014 | Whole-genome analysis in Korean patients with autoimmune myasthenia gravis | Consensus | Na S. | MG | EAS |
| Nakamura 2020 | 2020 | A multi-ethnic meta-analysis identifies novel genes, including ACSL5, associated with amyotrophic lateral sclerosis | Consensus | Nakamura R. | ALS | EAS, EUR |
| Nalls 2014 | 2014 | Large-scale meta-analysis of genome-wide association data identifies six new risk loci for Parkinson's disease | Consensus | Nalls M. | PD | EUR |
| Nalls 2019 | 2019 | Identification of novel risk loci, causal insights, and heritable risk for Parkinson's disease: a meta-analysis of genome-wide association studies | Consensus | Nalls M. | PD | EUR |
| Nazarian 2019 | 2019 | Genome-wide analysis of genetic predisposition to Alzheimer's disease and related sex disparities | Consensus | Nazarian A. | AD | EUR |
| Nicolas 2018 | 2018 | Genome-wide Analyses Identify KIF5A as a Novel ALS Gene | Consensus | Nicolas A. | ALS | EUR |
| Ordoñez 2015 | 2015 | Genomewide admixture study in Mexican Mestizos with multiple sclerosis | Consensus | Ordoñez G. | MS | AMR |
| Pankratz 2012 | 2012 | Meta-analysis of Parkinson's disease: identification of a novel locus, RIT2 | Consensus | Pankratz N. | PD | EUR |
| Park 2021 | 2021 | Novel Alzheimer's disease risk variants identified based on whole-genome sequencing of APOE E4 carriers | Consensus | Park J. | AD | EAS |
| Patsopoulos 2019 | 2019 | Multiple sclerosis genomic map implicates peripheral immune cells and microglia in susceptibility | Consensus | Patsopoulos N. | MS | EUR |
| Pérez-Palma 2014 | 2014 | Overrepresentation of glutamate signaling in Alzheimer's disease: network-based pathway enrichment using meta-analysis of genome-wide association studies | Consensus | Pérez-Palm E. | AD | EUR |
| Pottier 2018 | 2018 | Potential genetic modifiers of disease risk and age at onset in patients with frontotemporal lobar degeneration and GRN mutations: a genome-wide association study | Consensus | Pottier C. | FTD | EUR |
| Pottier 2019 | 2019 | Genome-wide analyses as part of the international FTLD-TDP whole-genome sequencing consortium reveals novel disease risk factors and increases support for immune dysfunction in FTLD | Consensus | Pottier C. | FTD | EUR |
| Real 2022 | 2022 | Association between the LRP1B and APOE loci in the development of Parkinson's disease dementia | Consensus | Real R. | PD | EUR |
| Reitz 2013 | 2013 | Variants in the ATP-Binding Cassette Transporter (ABCA7), Apolipoprotein E epsilon 4, and the Risk of Late-Onset Alzheimer Disease in African Americans | Consensus | Reitz C. | AD | AAC |
| Renton 2015 | 2015 | A genome-wide association study of myasthenia gravis | Consensus | Renton A. | MG | EUR |
| Reus 2021 | 2021 | Genome-wide association study of frontotemporal dementia identifies a C9ORF72 haplotype with a median of 12-G4C2 repeats that predisposes to pathological repeat expansions | Consensus | Reus L. | FTD | EUR |
| Rodrigo 2021 | 2021 | Imputation and Reanalysis of ExomeChip Data Identifies Novel, Conditional and Joint Genetic Effects on Parkinson's Disease Risk | Consensus | Rodrigo L. | PD | EUR |
| Rongve 2019 | 2019 | GBA and APOE E4 associate with sporadic dementia with Lewy bodies in European genome wide association study | Consensus | Rongve A. | LBD | EUR |
| Sakaue 2021 | 2021 | A cross-population atlas of genetic associations for 220 human phenotypes | Consensus | Sakaue S. | MG ,PD | EAS, EUR |
| Schwartzentruber 2021 | 2021 | Genome-wide meta-analysis, fine-mapping and integrative prioritization implicate new Alzheimer's disease risk genes | Consensus | Schwartzentruber J. | AD | EUR |
| Seldin 2016 | 2016 | Genome-Wide Association Study of Late-Onset Myasthenia Gravis: Confirmation of TNFRSF11A and Identification of ZBTB10 and Three Distinct HLA Associations | Consensus | Seldin M. | MG | EUR |
| Sherva 2014 | 2014 | Genome-wide association study of the rate of cognitive decline in Alzheimer's disease | Consensus | Sherva R. | AD | EUR |
| Sherva 2022 | 2022 | African ancestry GWAS of dementia in a large military cohort identifies significant risk loci | Consensus | Sherva R. | AD | AAC |
| Shigemizu 2021 | 2021 | Ethnic and trans-ethnic genome-wide association studies identify new loci influencing Japanese Alzheimer's disease risk | Consensus | Shigemizu D. | AD | EAS, EUR |
| Siitonen 2017 | 2017 | Genetics of early-onset Parkinson's disease in Finland: exome sequencing and genome-wide association study | Consensus | Siitonen A. | PD | EUR |
| Sims 2017 | 2017 | Rare coding variants in PLCG2, ABI3, and TREM2 implicate microglial-mediated innate immunity in Alzheimer's disease | Consensus | Sims R. | AD | EUR |
| Sorosina 2022 | 2022 | A multi-step genomic approach prioritized TBKBP1 gene as relevant for multiple sclerosis susceptibility | Consensus | Sorosina M. | MS | EUR |
| Steri 2017 | 2017 | Overexpression of the Cytokine BAFF and Autoimmunity Risk | Consensus | Steri M. | MS | EUR |
| Tan 2021 | 2021 | Genome-Wide Association Studies of Cognitive and Motor Progression in Parkinson's Disease | Consensus | Tan M. | PD | EUR |
| Tosto 2015 | 2015 | F-box/LRR-repeat protein 7 is genetically associated with Alzheimer's disease | Consensus | Tosto G. | AD | AAC |
| van Rheenen 2021 | 2021 | Common and rare variant association analyses in amyotrophic lateral sclerosis identify 15 risk loci with distinct genetic architectures and neuron-specific biology | Consensus | van Rheenen | ALS | EAS, EUR |
| Vandebergh 2021 | 2021 | Genetic Variation in WNT9B Increases Relapse Hazard in Multiple Sclerosis | Consensus | Vandebergh M. | MS | EUR |
| vanRheenen 2016 | 2016 | Genome-wide association analyses identify new risk variants and the genetic architecture of amyotrophic lateral sclerosis | Consensus | van Rheenen W. | ALS | EUR |
| Vardarajan 2018 | 2018 | Whole genome sequencing of Caribbean Hispanic families with late-onset Alzheimer's disease | Consensus | Vardarajan B. | AD | AAC |
| Wallen 2018 | 2018 | Plasticity-related gene 3 (LPPR1) and age at diagnosis of Parkinson disease | Consensus | Wallen Z. | PD | EUR |
| Wang 2015 | 2015 | Genetic Determinants of Survival in Patients with Alzheimer's Disease | Consensus | Wang X. | AD | EUR |
| Wang 2021 | 2021 | Similar Genetic Architecture of Alzheimer's Disease and Differential APOE Effect Between Sexes | Consensus | Wang H. | AD | EUR |
| Wei 2019 | 2019 | Identification of TYW3/CRYZ and FGD4 as susceptibility genes for amyotrophic lateral sclerosis | Consensus | Wei L. | ALS | EAS |
| Wightman 2021 | 2021 | A genome-wide association study with 1,126,563 individuals identifies new risk loci for Alzheimer's disease | Consensus | Wightman D. | AD | EUR |
| Witoelar 2018 | 2018 | Meta-analysis of Alzheimer's disease on 9,751 samples from Norway and IGAP study identifies four risk loci | Consensus | Witoelar A. | AD | EUR |
| Xie 2014 | 2014 | Genome-wide association study combining pathway analysis for typical sporadic amyotrophic lateral sclerosis in Chinese Han populations | Consensus | Xie T. | ALS | EAS |
| Yashin 2018 | 2018 | Hidden heterogeneity in Alzheimer's disease: Insights from genetic association studies and other analyses | Consensus | Yashin A. | AD | EUR |
