# Supplementary material for: Assessing the lack of diversity in genetics research across neurodegenerative diseases: A systematic review of the GWAS Catalog and literature
Source: Alzheimers Dement. 2024 Jun 21;20(8):5740–56. doi: 10.1002/alz.13873 (PMC11350004; doi:10.1002/alz.13873)
Supplement: Supplementary file 4 — Supporting Information [file ALZ-20-5740-s003.docx]

**Supplementary Table 3: Efforts to increase the diversity of genetic studies in NDDs.** This table lists resources for researchers who are interested in collecting and using diverse genetic samples.

| **Group** | **Abbreviation** | **Notable Aims** | **Website** |
| --- | --- | --- | --- |
| Multi-Partner Consortium to Expand Dementia Research in Latin America | ReDlat | leveraging connections with research communities in Latin America to grow a diverse database of dementia resources | <https://red-lat.com/> |
| Alzheimer’s Disease NeuroImaging Study | ADNI | growing more inclusive cell lines and generating partner data for multiple ancestrally diverse samples | <https://adni.loni.usc.edu/> |
| NIH’s Center for Alzheimer’s and Related Dementias | CARD | creating training materials, generating data to complement existing efforts, and supporting researchers in diverse communities. | <https://card.nih.gov/> |
| Genetic Architecture of Parkinson disease in India | GAP-India | plans to develop a large clinical/genomic biobank in India for PD | <https://pubmed.ncbi.nlm.nih.gov/32655481/> |
| Latin American Research Consortium on the Genetics of PD | LARGE-PD | address inclusivity and genomic differences within and across Latino populations in Parkinson’s disease | <https://large-pd.org/> |
| Global Parkinson’s Genetics Program | GP2 | *genotype >150,000 individuals from around the world in order to better understand Parkinson’s disease  *increase diversity in the investigators making use of the data, providing training and resources to ensure an open and equal field of play | <https://gp2.org/> |
| Black and African Americans Connections to Parkinson’s Disease Study (GP2 Funded) | BLAACPD | assess the genetic architecture of PD of Black and African Americans | <https://www.blaacpd.org/DJYD/> |
| European DLB consortium | E-DLB | study dementia with Lewy Bodies (DLB) and collect longitudinal, clinical, and biomarker data from 20 countries in Europe, Australia, US, Japan, Columbia, and China | <https://www.e-dlb.com/> |
| Northeast Amyotrophic Lateral Sclerosis Consortium | NEALS | study amyotrophic lateral sclerosis (ALS) and motor neuron disease in more than 130 sites, including centers in Japan, Israel, Mexico, Canada, Australia, and Italy | <https://neals.org/> |
| European Network to Cure ALS | ENCALS | study amyotrophic lateral sclerosis (ALS) and motor neuron disease in Europe | <https://www.encals.eu/> |
| Pan-Asian Consortium for Treatment and Research in ALS | PACTALS | study amyotrophic lateral sclerosis (ALS) and motor neuron disease in the Asia-Pacific region. | <https://pactals.org/> |
